# Compassionate Use of Avacopan in Difficult-to-Treat Antineutrophil Cytoplasmic Antibody–Associated Vasculitis

**DOI:** 10.1016/j.ekir.2021.11.036

**Published:** 2021-12-08

**Authors:** Jolijn R. van Leeuwen, Obbo W. Bredewold, Laura S. van Dam, Stella L. Werkman, Jacqueline T. Jonker, Miranda Geelhoed, Antonius P.M. Langeveld, Hilde H.F. Remmelts, Maud M. van den Broecke, Argho Ray, Ton J. Rabelink, Y.K. Onno Teng

**Affiliations:** 1 Center of Expertise for Lupus-, Vasculitis- and Complement-mediated Systemic diseases (LuVaCs), Department of Internal Medicine—Nephrology Section, Leiden University Medical Center, Leiden, The Netherlands; 2 Department of Nephrology, Alrijne Ziekenhuis, Leiden, The Netherlands; 3 Center of Expertise for Lupus-, Vasculitis- and Complement-mediated Systemic diseases (LuVaCs), Department of Pulmonology, Leiden University Medical Center, Leiden, The Netherlands; 4 Center of Expertise for Lupus-, Vasculitis- and Complement-mediated Systemic diseases (LuVaCs), Department Otorhinolaryngology, Leiden University Medical Center, Leiden, The Netherlands; 5 Department of Nephrology, Meander Medisch Centrum, Amersfoort, The Netherlands

## Introduction

Avacopan is a new, promising treatment for antineutrophil cytoplasmic antibody–associated vasculitis (AAV) and can potentially, significantly reduce steroid use. There were 2 phase 2 trials^1^^,^^2^ and 1 phase 3 trial^3^ which concluded that avacopan was safe with no higher incidence of adverse events in patients with AAV. Compared with steroids added to standard immunosuppression with cyclophosphamide or rituximab, avacopan was found to have noninferiority for treatment response at 12 weeks^1^ and 26 weeks.^3^ Avacopan proved superior for sustaining remission at 52 weeks and reducing relapses within the first 52 weeks from 21% to 10% compared with a prednisone tapering schedule of 20 weeks.^3^ Patients using avacopan had greater improvement on health-related quality of life,^1^, ^2^, ^3^ which was likely related to avoidance of steroid-related adverse effects.^2^^,^^3^

Most recently, avacopan was approved for the treatment of AAV by the US Food and Drug Administration and approval is recommended by a committee of the European Medicines Agency to the European Commission.^4^^,^^5^ In addition to currently available data of randomized trials, we now report the clinical experience with avacopan in difficult-to-treat patients with AAV in the setting of a compassionate use program.

## Results

### Cases

A total of 8 adult patients with granulomatosis with polyangiitis and microscopic polyangiitis were treated within the avacopan compassionate use program at our institute. Patient and AAV-relevant characteristics are summarized in Table 1. In 4 cases (1–4), the indication to apply for avacopan was refractory disease with steroid resistance, characterized by continuous or worsening disease despite recent induction therapy with high-dose steroids. In 2 cases, the indication was steroid dependence owing to relapsing (case 5) or grumbling (case 6) disease when prednisone was reduced <15 mg per day. Furthermore, 2 cases (7, 8) started avacopan because of necessity to avoid steroid-related toxicity based on the patient’s medical history with obesity and/or diabetes and previous severe steroid-related toxicity. Briefly, 6 patients had generalized disease and 2 patients had ear, nose, and throat–limited disease at the start of avacopan. Typically, the patients had relapsing disease (numbers of flares ranging from 0 to 3) and received multiple previous remission-induction therapies (ranging from 1 to 6) as specified in Table 1. The median time between the start of latest induction therapy and start of avacopan was 7.7 (0.4–15.9) weeks coinciding with a median supply time of avacopan of 5.5 (2.9–7.9) weeks. During this time, all patients received prednisone as a bridge to avacopan initiation. Concomitant to avacopan, 4 patients (cases 3, 5, 6, 7) received maintenance treatment with rituximab and 1 patient received rituximab induction therapy.

**Table 1:** Patient, AAV, and treatment characteristics

	Case 1	Case 2	Case 3	Case 4	Case 5	Case 6	Case 7	Case 8
**Before start avacopan**								
Age	54	67	27	37	38	54	23	65
Sex	M	M	F	M	F	F	M	M
ANCA serology	MPO	MPO	PR3	MPO	PR3	PR3	PR3	MPO
Organ involvement	Renal Pulmonary	Renal Pulmonary Skin	ENT	Renal	Pulmonary ENT	ENT Joints	Renal Pulmonary ENT Joints Skin	Renal Neurologic
Duration of vasculitis in months	6	139	19	15	19	32	37	100
Number of flares	0	3	1	0	0	0	1	1
Number of induction therapies	2	6	2	3	3	1	2	2
Previous immunosuppressive medication	−1 mo: CYC −6 mo: MP, RTX	−4 mo: MP, PE, obinutuzumab −5 mo: MP, PE, obinutuzumab −1 yr: RTX, MP, CYC −11/9/7 yr: MMF	−3 mo: MP, RTX −2 yr: MP, RTX	−2 mo: MP, RTX, AZA −7 mo: RTX −1 yr: MP, CYC	−1 mo: MP, CYC −4 mo: RTX −1 yr: CYC	−2 mo RTX −6 mo RTX, MMF −8 mo: AZA −1 yr: RTX −2 to 3 yr: RTX, MTX	−1 mo: RTX −3 yr: MP, RTX, AZA	−1 mo: MP, RTX −8 yr: CYC
Anti-CD20 cumulative doses in mg	2000	4000	4000	2500	2000	6000	4000	2000
CYC cumulative doses in mg	3000	1500	0	12,000	17,000	0	0	22000
Solumedrol cumulative doses in mg	3000	9000	6000	4500	3000	0	3000	3000
**After start avacopan**								
Indication to start	Steroid resistance	Steroid resistance	Steroid resistance	Steroid resistance	Steroid dependence	Steroid dependence	Steroid-related toxicity	Steroid-related toxicity
Supply time (wk)	3.6	6.9	6.6	6.0	7.9	5.0	3.6	2.9
Prednisone at start (mg/d)	25	5	20	20	5	15	20	30
BVAS at start	11	0	3	3	2	2	15	5
Concomitant maintenance treatment	Prednisone (5 mg/d)	Prednisone (2.5 mg/d)	+2 mo RTX (1000 mg) +8 mo RTX (500 mg)		+14 mo: RTX (1000 mg)	Prednisone (7.5 mg/d) +1 yr: RTX (1000 mg)	+1 yr: RTX (500 mg)	
Extra induction therapies					+8 mo RTX (2000 mg)			

ANCA, antineutrophil cytoplasmic antibody; AZA, azathioprine; BVAS, Birmingham vasculitis activity score; CYC, cyclophosphamide; ENT, ear, nose, throat; F, female; PR3, proteinase 3; M, male; MMF, mycophenolate mofetil; MP, methylprednisolone; MPO, myeloperoxidase; MTX, methotrexate; N/A, not applicable; PE, plasma exchange; RTX, rituximab.

### Treatment Responses and Steroid-Related Toxicity

**Figure 1:**
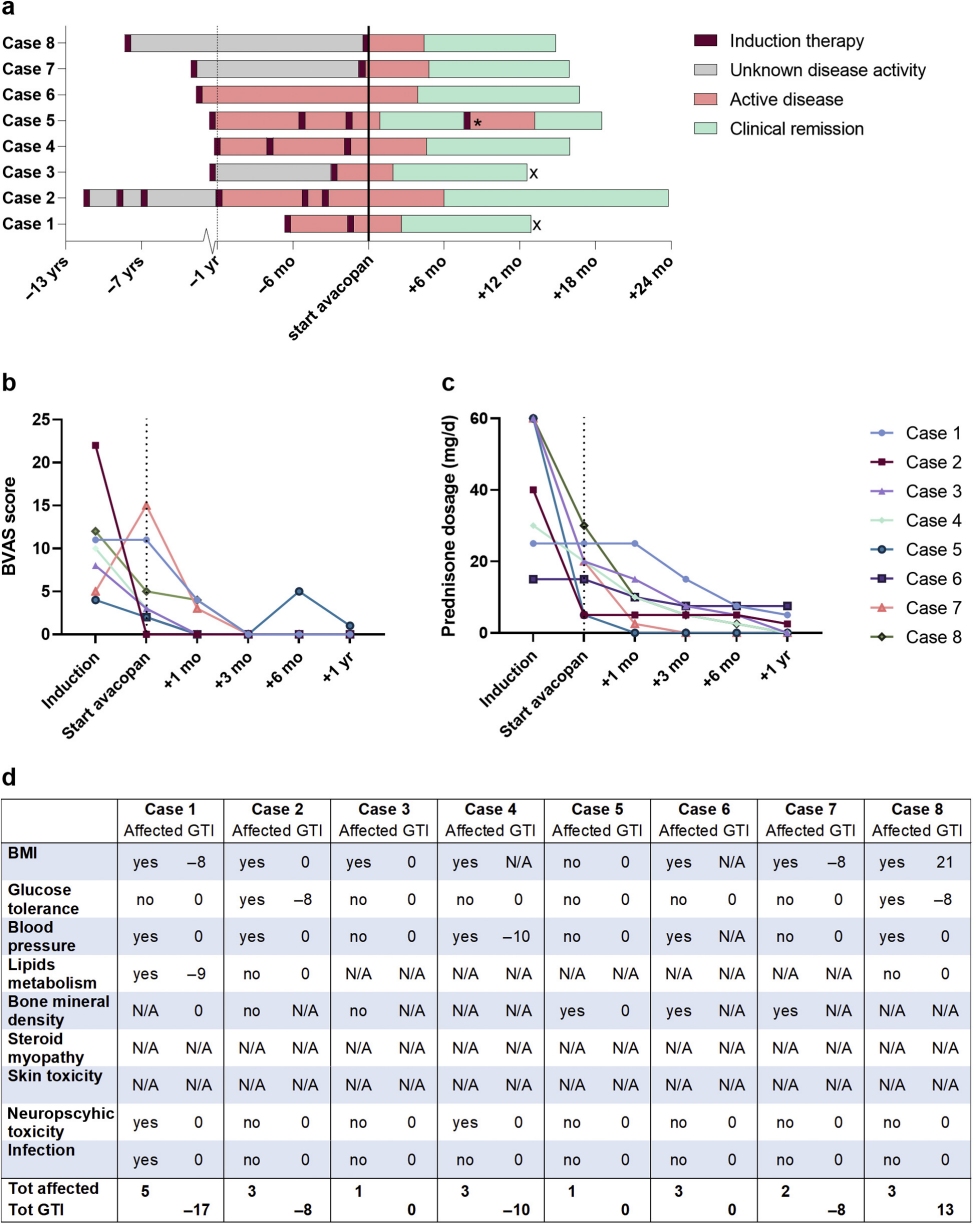
Treatment outcomes. (a) Disease course per patient in relation to start of avacopan (black line). Columns start at moment of diagnoses and end at current date or stop of avacopan (x). Note that the x-as changes at −1 yr (dotted line) from 6 yr to 6 mo per thick. For some periods of time, it could not be reconstructed if or when remission was achieved (unknown disease activity). ∗Reduction of avacopan dosing to 20 mg twice a day. (b) BVAS per patient at different time points. (c) Prednisone dosage in mg/d per patient at different time points. (d) Composite items of GTI. Per patient is revealed when the item was affected at the start of avacopan and the GTI index is scored after 1 yr of avacopan use. Scores can range from −36 to 439, with increasing scores relating to an increase in glucocorticoid toxicity burden and negative scores reflecting an improvement in toxicity. Last row is total affected items and total GTI score. BMI, body mass index; BVAS, Birmingham vasculitis activity score; GTI, Glucocorticoid Toxicity Index; mo, month; N/A, not applicable; Tot, total; yr, year.

Disease courses of individual patients before and after the initiation of avacopan are depicted in Figure 1a illustrating frequent severe relapses requiring remission-induction treatment before avacopan was initiated. This contrasted with the time after avacopan was started: all patients achieved clinical remission within 6 months and accordingly the Birmingham vasculitis activity score returned to 0 in all patients (Figure 1b). Noteworthy, only 1 patient (case 5) experienced a major flare with pulmonary involvement 6 months after avacopan start. The event coincided with a reduction of avacopan dosing to 20 mg twice a day which was necessary owing to delayed supply of avacopan related to transport restrictions during the second wave in the COVID-19 pandemic. The patient’s flare was successfully managed with i.v. rituximab, a single intra-articular steroid injection for arthritis of the knee, and reinstitution of avacopan 30 mg twice a day without the additional use of oral steroids. With respect to renal involvement in 5 of 8 patients, estimated glomerular filtration rate ranged from 32 to 90 ml/min at start of avacopan and slightly improved in 4 patients (range +5 to +9 ml/min) and decreased in none. Noteworthy, longstanding hematuria in case 2 disappeared (<18/ml) after 4 months of avacopan treatment.

As found in Figure 1c, steroid tapering was successful in all patients with 5 discontinuing prednisone and 3 (cases 1, 2, 6) using low-dose prednisone (2.5–7.5 mg/d). With respect to steroid-related toxicity effects, as defined by the Glucocorticoid Toxicity Index (GTI),^6^ patients had a median of 3 (1–5) affected items at avacopan start (Figure 1d). After 1 year of avacopan use, the GTI improved in 4 patients related to improvement of body mass index, glucose tolerance, blood pressure, or lipid metabolism, and GTI remained stable in 3 patients. In 1 patient (case 8), GTI worsened related to weight gain despite improved glucose intolerance. Noteworthy, 2 patients (cases 1 and 4) had steroid-related depressive symptoms which improved in both patients on avacopan and 1 patient (case 1) could stop antidepressant therapy.

Even though, during compassionate use, adverse events are not structurally registered, no adverse events, side effects, or infections related to avacopan were reported. There were 6 patients who are currently satisfactorily continuing avacopan. Case 1 participated in the initial stages of the compassionate use program in which avacopan use was allowed for 1 year only. Case 3 stopped avacopan because of a pregnancy wish.

## Discussion

This study describes real-life practice data on the compassionate use of avacopan in difficult-to-treat patients with AAV. Avacopan contributed to achieving and maintaining remission in these patients with AAV while breaking through steroid dependency and allowing steroid reductions. Obviously, it is impossible to determine the clinical efficacy of avacopan owing to previous and concomitant intensive remission-induction immunosuppression and concomitant rituximab maintenance treatment in 4 patients. Nevertheless, avacopan had beneficial and added value in the treatment of our difficult-to-treat AAV cases with respect to improved disease control and reduced steroid-related toxicity.

During the avacopan compassionate use program at our center, we applied for avacopan in cases 1 to 4 because of insufficient response to previous intensive remission-induction therapy, including high-dose steroids. All 4 patients achieved clinical and persistent remission on avacopan initiation. Furthermore, cases 5 and 6 achieved full remission for the first time since their diagnoses during avacopan treatment, in contrast to previous continuous active disease with steroid dependence. Noteworthy, in 1 patient (case 5), we experienced that reinstitution of avacopan successfully diverted a major disease flare. The latter is corroborated by early phase 1 study data revealing a dose-related, pharmacologic C5a-R inhibition.^7^

With respect to steroid use, only 3 of 8 patients required prednisone in a low dosage after 1 year of avacopan treatment. In addition, in 2 cases of steroid dependency, steroids could not be tapered <15 mg/d. With avacopan treatment, steroids were fully tapered to 0 in 1 patient (case 5) and a clinically relevant reduction to 7.5 mg/d in the second patient (case 6). Taken together, compassionate-use avacopan allowed to ameliorate disease and to significantly reduce and stop steroid use in difficult-to-treat patients with AAV while improvement of steroid-related toxicity was observed.

Last, this study on a case series of compassionate-use avacopan provides guidance to future observational studies with avacopan. The challenge to identify beneficial effects of avacopan in clinical data of patients with AAV is defined by the absence of disease activity, disease flares, steroids, and steroid-related toxicity. It requires careful considerations to firmly determine benefit by proving the absence of clinically relevant events that physicians automatically strive for in routine clinical practice. Furthermore, it will remain a significant challenge to prove the clinical efficacy of avacopan on the background of highly intensive immunosuppression necessary for remission induction in AAV. Thus, to further investigate the potential benefits of avacopan in patients with AAV in observational studies, we emphasize assessing accurate medical histories with emphasis on disease courses, steroid dosing, steroid-related toxicity using validated GTI scores, and disease-relevant patient-reported outcomes.

In conclusion, we here provide the first real-life practice observations on the compassionate use of avacopan in difficult-to-treat patients with AAV. Our study describes the clinical added value of avacopan in AAV treatment and that beneficial effects of avacopan are predominantly determined by the absence of adverse events, such as persistent disease activity, steroid dependence, and steroid-related toxicity.

## Disclosure

The work of YKOT was supported by the Dutch Kidney Foundation (17OKG04) and by the Arthritis Research and Collaboration Hub Foundation. Arthritis Research and Collaboration Hub is funded by Dutch Arthritis Foundation (ReumaNederland). YKOT received an unrestricted research grant and consultancy fees from Vifor Pharma. All the other authors declared no competing interests.

## Supplementary Material

**Supplementary Methods.**

**STROBE Statement** (PDF)
