# Supplementary material for: Compassionate Use of Avacopan in Difficult-to-Treat Antineutrophil Cytoplasmic Antibody–Associated Vasculitis
Source: Kidney Int Rep. 2021 Dec 8;7(3):624–8. doi: 10.1016/j.ekir.2021.11.036 (PMC8897689; doi:10.1016/j.ekir.2021.11.036)
Supplement: Supplementary File (PDF) [file mmc1.pdf]

Supplementary Material:

1. Supplementary Methods

2. STROBE Checklist

1. Supplementary Methods

Since 2014 avacopan has been granted orphan designation by the European Medicines Agency for treatment of AAV. In this article we describe adult patients with myeloperoxidase (MPO) or proteinase 3 (PR3) positive AAV who were treated with avacopan in the setting of a compassionate use program at the department of Nephrology of the Leiden University Medical Center since 2017. Before entering the global compassionate use program each patient application had to be approved by the Dutch Ministry of Health requiring a declaration of the absence of an adequate drug alternative and an urgent request by the treating physician declaring that the condition could not be adequately treated with medicines currently approved in the Netherlands.

As part of a global compassionate use program eligibility criteria were defined by the Chemocentryx/Vifor Pharma, as patients with a) a confirmed diagnosis of organ or life-threatening granulomatosis with polyangiitis (GPA) or microscopic polyangiitis (MPA) who required therapy for induction treatment to achieve remission (new presentation or relapsing disease) from organ or life-threatening GPA or MPA; and b) a high risk of developing a glucocorticoid complication in particular new onset diabetes mellitus (high BMI, family history), bone complication (past history or bone densitometry) or a past medical history of condition likely to be exacerbated with high dose glucocorticoids - diabetes, cardiac failure or severe coronary arterial disease, bone complication, previous severe mental health adverse event with high dose glucocorticoids requiring dose reduction or cessation; and c) is not of child bearing potential or if childbearing age commits to use approved birth control. Non-eligibility criteria were defined as patients who a) were younger than 18 years; b) had another form of vasculitis than GPA or MPA; c) had acute hepatitis (including hepatitis A) or chronic hepatitis B, hepatitis C or HIV disease; d) had evidence of hepatic disease - AST, ALT, alkaline phosphatase greater than 3 times upper limit of normal or bilirubin greater than 2 times upper limit of normal; e) had any other known multisystem autoimmune disease; f) had alveolar hemorrhage requiring invasive pulmonary ventilator support; g) had a kidney transplant; h) had a white blood cell count less than 3500/µL, or neutrophil count less than 1500/µL, or lymphocyte count less than 500/µL before start of avacopan; i) were pregnant or breast feeding; j) were taking a strong inducer of the cytochrome P450 (CYP3A4) enzyme such as carbamazepine, phenobarbital, rifampicin or St John’s wort; k) experienced any of the following within 12 weeks of consideration of the program – symptomatic heart failure requiring prescription medication, unstable angina (unless successfully treated with stent or bypass surgery), clinically significant cardiac arrhythmia, myocardial infarction or stroke; l) had a clinically significant abnormal ECG; m) had an estimated glomerular filtration rate < 15 mL/minute/1.73 m2 (using the MDRD method); n) had a known

hypersensitivity to avacopan or inactive ingredients of the avacopan capsules (including gelatin, polyethylene glycol, or Cremophor).

For all patients, disease relevant characteristics before and during avacopan treatment were collected including gender, age, organ involvement, duration of vasculitis and number of flares at baseline, medication history and relevant laboratory results. Patients were classified by the authors to their reason to start avacopan. This was steroid resistance, defined as continuous or worsening disease despite induction therapy with steroids, steroid dependence, defined as grumbling or relapsing disease without the possibility to taper steroids below 10mg/day, or steroid toxicity, defined as the necessity to avoid high-risk side effects. Clinical remission was based on physician's clinical assessments as reported in the electronic health records (EHC).

Additionally, we collected relevant data to assess steroid-related toxicity effects in line with the Glucocorticoid Toxicity Index (GTI) (v2017), including infectious adverse events. The GTI is a tool to quantify toxic effect of steroids over a time interval with a scoring system on nine items of a composite list.[5] For each item we objectified if it was affected at baseline and scored the change according to the GTI tool after one year of avacopan use.

STROBE (Strengthening The Reporting of OBservational Studies in Epidemiology) Checklist

A checklist of items that should be included in reports of observational studies. You must report the page number in your manuscript where you consider each of the items listed in this checklist. If you have not included this information, either revise your manuscript accordingly before submitting or note N/A.

Note: An Explanation and Elaboration article discusses each checklist item and gives methodological background and published examples of transparent reporting. The STROBE checklist is best used in conjunction with this article (freely available on the Web sites of PLoS Medicine at http://www.plosmedicine.org/, Annals of Internal Medicine at http://www.annals.org/, and Epidemiology at http://www.epidem.com/). Information on the STROBE Initiative is available at www.strobe-statement.org.

Section and Item	Item No.	Recommendation	Reported on Page No.
**Title and Abstract**	1	(*a*) Indicate the study's design with a commonly used term in the title or the abstract	1
		(*b*) Provide in the abstract an informative and balanced summary of what was done and what was found	N/A
**Introduction**			
Background/Rationale	2	Explain the scientific background and rationale for the investigation being reported	2
Objectives	3	State specific objectives, including any prespecified hypotheses	2
**Methods**			
Study Design	4	Present key elements of study design early in the paper	2
Setting	5	Describe the setting, locations, and relevant dates, including periods of recruitment, exposure, follow-up, and data collection	S1, p3
Participants	6	(*a*) *Cohort study*—Give the eligibility criteria, and the sources and methods of selection of participants. Describe methods of follow-up	S1, p2
		Case-control study—Give the eligibility criteria, and the sources and methods of case ascertainment and control selection. Give the rationale for the choice of cases and controls	N/A
		Cross-sectional study—Give the eligibility criteria, and the sources and methods of selection of participants	N/A
		(*b*) *Cohort study*—For matched studies, give matching criteria and number of exposed and unexposed	N/A
		Case-control study—For matched studies, give matching criteria and the number of controls per case	N/A
Variables	7	Clearly define all outcomes, exposures, predictors, potential confounders, and effect modifiers. Give diagnostic criteria, if applicable	S1, p3

Section and Item	Item No.	Recommendation	Reported on Page No.
Data Sources/ Measurement	8*	For each variable of interest, give sources of data and details of methods of assessment (measurement). Describe comparability of assessment methods if there is more than one group	S1, p3
Bias	9	Describe any efforts to address potential sources of bias	N/A
Study Size	10	Explain how the study size was arrived at	2
Quantitative Variables	11	Explain how quantitative variables were handled in the analyses. If applicable, describe which groupings were chosen and why	N/A
Statistical Methods	12	(*a*) Describe all statistical methods, including those used to control for confounding	N/A
		(*b*) Describe any methods used to examine subgroups and interactions	N/A
		(*c*) Explain how missing data were addressed	N/A
		(*d*) *Cohort study*—If applicable, explain how loss to follow-up was addressed	N/A
		Case-control study—If applicable, explain how matching of cases and controls was addressed	N/A
		Cross-sectional study—If applicable, describe analytical methods taking account of sampling strategy	N/A
		(*e*) Describe any sensitivity analyses	N/A
**Results**			
Participants	13*	(a) Report numbers of individuals at each stage of study—eg numbers potentially eligible, examined for eligibility, confirmed eligible, included in the study, completing follow-up, and analysed	2
		(b) Give reasons for non-participation at each stage	N/A
		(c) Consider use of a flow diagram	N/A
Descriptive Data	14*	(a) Give characteristics of study participants (eg demographic, clinical, social) and information on exposures and potential confounders	9 (table 1)
		(b) Indicate number of participants with missing data for each variable of interest	N/A
		(c) *Cohort study*—Summarise follow-up time (eg, average and total amount)	N/A
Outcome Data	15*	*Cohort study*—Report numbers of outcome events or summary measures over time	2
		Case-control study—Report numbers in each exposure category, or summary measures of exposure	N/A
		Cross-sectional study—Report numbers of outcome events or summary measures	N/A

Section and Item	Item No.	Recommendation	Reported on Page No.
Main Results	16	(*a*) Give unadjusted estimates and, if applicable, confounder-adjusted estimates and their precision (eg, 95% confidence interval). Make clear which confounders were adjusted for and why they were included	3
		(*b*) Report category boundaries when continuous variables were categorized	N/A
		(*c*) If relevant, consider translating estimates of relative risk into absolute risk for a meaningful time period	N/A
Other Analyses	17	Report other analyses done—eg analyses of subgroups and interactions, and sensitivity analyses	3
**Discussion**			
Key Results	18	Summarise key results with reference to study objectives	4
Limitations	19	Discuss limitations of the study, taking into account sources of potential bias or imprecision. Discuss both direction and magnitude of any potential bias	4
Interpretation	20	Give a cautious overall interpretation of results considering objectives, limitations, multiplicity of analyses, results from similar studies, and other relevant evidence	4
Generalisability	21	Discuss the generalisability (external validity) of the study results	4
**Other Information**			
Funding	22	Give the source of funding and the role of the funders for the present study and, if applicable, for the original study on which the present article is based	6

*Give information separately for cases and controls in case-control studies and, if applicable, for exposed and unexposed groups in cohort and cross-sectional studies.

Once you have completed this checklist, please save a copy and upload it as part of your submission. DO NOT include this checklist as part of the main manuscript document. It must be uploaded as a separate file.
